# Recent Trends in Improving the Oxidative Stability of Oil-Based Food Products by Inhibiting Oxidation at the Interfacial Region

**DOI:** 10.3390/foods12061191

**Published:** 2023-03-11

**Authors:** Malihe Keramat, Elham Ehsandoost, Mohammad-Taghi Golmakani

**Affiliations:** Department of Food Science and Technology, School of Agriculture, Shiraz University, Shiraz 71441-65186, Iran

**Keywords:** bulk oil, emulsion, interfacial region, oxidation

## Abstract

In recent years, new approaches have been developed to limit the oxidation of oil-based food products by inhibiting peroxidation at the interfacial region. This review article describes and discusses these particular approaches. In bulk oils, modifying the polarity of antioxidants by chemical methods (e.g., esterifying antioxidants with fatty alcohol or fatty acids) and combining antioxidants with surfactants with low hydrophilic–lipophilic balance value (e.g., lecithin and polyglycerol polyricinoleate) can be effective strategies for inhibiting peroxidation. Compared to monolayer emulsions, a thick interfacial layer in multilayer emulsions and Pickering emulsions can act as a physical barrier. Meanwhile, high viscosity of the water phase in emulsion gels tends to hinder the diffusion of pro-oxidants into the interfacial region. Furthermore, applying surface-active substances with antioxidant properties (such as proteins, peptides, polysaccharides, and complexes of protein-polysaccharide, protein-polyphenol, protein-saponin, and protein-polysaccharide-polyphenol) that adsorb at the interfacial area is another novel method for enhancing oil-in-water emulsion oxidative stability. Furthermore, localizing antioxidants at the interfacial region through lipophilization of hydrophilic antioxidants, conjugating antioxidants with surfactants, or entrapping antioxidants into Pickering particles can be considered new strategies for reducing the emulsion peroxidation.

## 1. Introduction

Lipid oxidation is an important economic concern in the food industry, primarily because it can influence the quality attributes and nutritional profile of oil-based products. Food manufacturers often have different options to use different methods and reduce the oxidation rate of oil-based food products. One such method is replacing polyunsaturated fatty acids (PUFAs) with fats that comprise high concentrations of saturated fatty acids. However, this method usually reduces the nutritional profile of oil-based food products and, at the same time, increases the risk of cardiovascular disease. Another approach that can be helpful in reducing lipid oxidation is the partial hydrogenation of oils and fats that can contribute to the conversion of PUFA—types that are highly susceptible to lipid oxidation—to more saturated fatty acids. However, trans-fatty acids are formed during partial hydrogenation [1]. Therefore, researchers in the food industry pay substantial attention to developing new strategies that can assist in inhibiting lipid oxidation in food products with rich PUFA content.

Bulk oils contain surface agents such as monoacylglycerols, diacylglycerols, phospholipids, sterols, and free fatty acids, as well as oxidation products that are generated through lipid oxidation. Furthermore, bulk oils contain small amounts of water. Surface-active agents can produce different association colloids, such as microemulsions, reverse micelles, cylindrical aggregates, and lamellar structures in the presence of water. Surface-active lipid hydroperoxides can aggregate at the interfacial region of association colloids and be decomposed by water-soluble metal ions into free radicals, thereby further increasing the rate of lipid oxidation [2]. Incorporating higher amounts of antioxidant molecules into the interfacial region of association colloids can significantly reduce the oxidation of bulk oils [3]. For instance, Keramat et al. [4] reported that sunflower oil containing a combination of sesamol and polyglycerol polyricinoleate showed higher oxidative stability than that of sunflower oil containing sesamol alone. This is related to the better interfacial performance of sesamol in the presence of polyglycerol polyricinoleate.

In the case of oil-in-water (O/W) emulsions, it has been proposed that oxidation occurs at the interfacial area where PUFAs in the oil phase contact with pro-oxidants in the water phase [2]. 

Despite remarkable focus on free radical chain mechanisms in the available literature, there is not much information on interfacial phenomena that contribute to lipid oxidation in bulk oils [3]. In O/W emulsions, the thickness, charge, and composition of the interface are the most important factors affecting the oxidation rate at the interfacial region. A large number of studies have demonstrated success in inhibiting PUFA oxidation by manipulating the properties of the interfacial region [5]. For instance, Meng et al. [6] altered the microstructure of O/W emulsion to reduce lipid oxidation. They observed that emulsions produced by zein/carboxy methyl dextrin nanoparticles showed a lower oxidation rate than those emulsions produced by zein nanoparticles.

This paper describes and discusses novel approaches that have been developed in recent years to reduce lipid oxidation at the interfacial region of oil-based food products. After briefly describing the oxidation mechanism in bulk oil and O/W emulsions, this paper suggests efficient strategies for designing lipid systems with high oxidative stability.

## 2. Lipid Oxidation Mechanism in the Oil-Based Food Products

### 2.1. Bulk Oil 

Bulk oil is a heterogeneous system that comprises small levels of water. In addition, bulk oil contains various amphiphilic compounds that can entrap water molecules and produce reverse micelles in the bulk oil. These structures, known as association colloids, are primarily lamellar structures and reverse micelles. Various types of water or concentrations of surface-active compounds can result in various shapes of association colloids and, thus, impact lipid oxidation in different ways [7]. Some surface-active compounds have low values of hydrophilic–lipophilic balance, namely monoacylglycerol (~3.4–3.8), diacylglycerol (~1.8), and free fatty acids (~1). These compounds are capable of creating reverse micelles in bulk oil. Phospholipids with a medium hydrophilic–lipophilic balance value (~8) can create reverse micelles and lamellar structures in bulk oil. Lipid hydroperoxides can reportedly accumulate at the interfacial region of reverse micelles [8].

#### 2.1.1. Role of Association Colloids on Bulk Oil Peroxidation

Association colloids can affect oxidation rate because they can produce oil–water interfaces where antioxidants and pro-oxidant compounds react with triacylglycerols [2]. A list of possible pro-oxidants in lipid systems is presented in Table 1. It is reported that phosphatidylcholine (PC) and phosphatidylethanolamine (PE) act as pro-oxidants in stripped peanut oil when their concentrations are beyond the critical micelle concentration (CMC). This pro-oxidant activity can be attributed to the presence of oil–water interfaces produced by phospholipid reverse micelles that can alter the location of amphiphilic and hydrophilic pro-oxidant compounds so that they approach the triacylglycerol substrate. In addition, phospholipids can decrease oil surface tension, which results in an enhanced oxygen diffusion rate in the oil [9]. It has been shown that free fatty acids can affect the reverse micelle structure of DOPC (1,2-dioleoyl-sn-glycero-3-phosphocholine) in bulk oil. Oxidation experiments on lipid hydroperoxides and an evaluation of hexanal production showed that free fatty acids exhibit pro-oxidant effects in the absence or presence of DOPC. Free fatty acids can bind metal ions, render them more pro-oxidative, and accelerate the decomposition of lipid hydroperoxides. In addition, free fatty acids can decrease surface tension and enhance the diffusion rate of oxygen from the headspace into bulk oil [10]. The association colloids formed by mixtures of surface-active compounds (dioleoylphosphatidylethanolamine, 1,2-dioleoyl-sn-glycero-3-phosphocholine, diolein, stigmasterol, and oleic acid) increase the lipid oxidation rate in a mixture of stripped corn oil/medium chain triglycerides [11]. The fatty acid profile can affect the properties of association colloids and the oxidation rate of bulk oils. Homma et al. [12] reported that the presence of association colloids in stripped high-oleic safflower oil and high-linoleic safflower oil did not have an effect on the lag phases of lipid hydroperoxides, nonanal, or hexanal formation. However, association colloids reduced the lag phases of both lipid hydroperoxides and propanal formation in fish triacylglycerols. Jo and Lee [13] reported that propanal and hexanal enhanced the CMC of DOPC, while the CMC of DOPC decreased in the presence of nonanal. These observations indicate that aldehydes participate in producing association colloids in bulk oils. Furthermore, they stated that aldehydes could accelerate the rate of oxidation as they participate in the production of more association colloids. 

#### 2.1.2. Effect of Association Colloids on the Efficiency of Antioxidants

Association colloids can impact the efficiency of antioxidant compounds in bulk oil [20]. For instance, it has been reported that DOPC-reverse micelles improve the efficiency of Trolox (6-Hydroxy-2,5,7,8-tetramethylchroman-2-carboxylic acid) and α-tocopherol when applied at a low concentration (10 µM); however, their efficiency decreased at a high concentration (100 µM). In addition, hydrophobic α-tocopherol was less efficient than hydrophilic Trolox. This difference in the efficiency of α-tocopherol and Trolox can be attributed to the differences in their physical location on the DOPC-reverse micelles [7]. In addition, when α-tocopherol and canolol were used at concentrations of 100 µM in rapeseed oil, their antioxidant efficiency decreased in the presence of DOPC association colloids during the storage period. This decrease in antioxidant efficiency could be related to the formation of amphiphilic compounds during autoxidation, which can impact the structure of reverse micelles and modify antioxidant efficiency [21]. According to Laguerre et al. [20], the efficiency of hexadecyl chlorogenate decreased when DOPC was incorporated into corn oil at a concentration higher than its CMC (200 µM). In contrast, DOPC did not alter the efficiency of chlorogenic acid. On the contrary, the antioxidant efficiency of chlorogenic acid decreased when a combination of DOPC (200 µM) and water (∼400 ppm) was incorporated into the corn oil. However, a combination of DOPC and water did not alter the efficiency of hexadecyl chlorogenate. Adding water to stripped corn oil containing DOPC produced reverse micelles with a water core. This water core can enhance the affinity of free chlorogenic acid for locating into the reverse micelles, where it can interact with water-soluble metal ions or metal ions attached to anionic phospholipids, thereby enhancing the pro-oxidant effect of the transition metals [20]. 

### 2.2. O/W Emulsion

**Figure 1:**
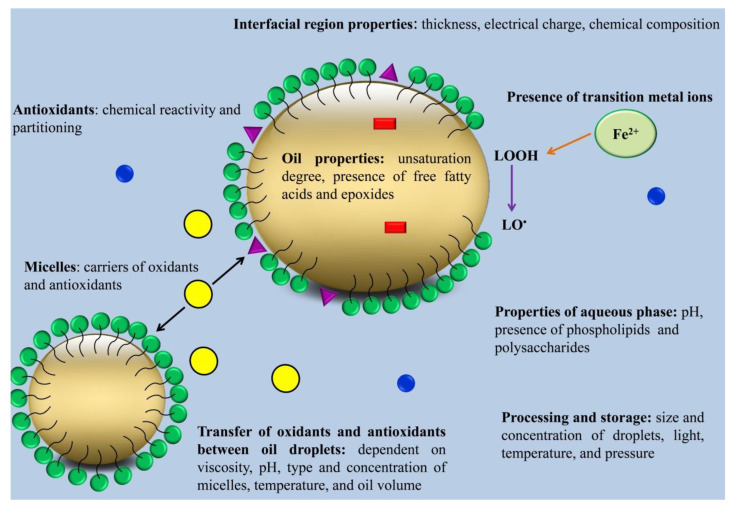
Factors affecting O/W emulsion oxidation (hydrophobic antioxidants: red rectangle; hydrophilic antioxidants: blue circle; amphiphilic antioxidants: purple triangle; and micelles: yellow circle ) (adopted from Ref. [27]).

O/W emulsions usually oxidize faster than bulk oils. The faster oxidation rate of O/W emulsions is related to several reasons. First, the presence of the interfacial region between the water and oil phase is assumed to promote contact between pro-oxidant compounds present in the water phase and unsaturated lipids. In addition, a high interfacial region could enhance the accessibility of the oil phase to the oxygen in the water phase. Second, the process of emulsion production can enhance oxidation by incorporating oxygen, the direct formation of free radicals via acoustic cavitation in the case of sonication or by overheating due to shear stress [22,23,24]. The physicochemical properties of the interfacial region, transfer of oxidants and antioxidants between oil droplets, lipid and aqueous phase components, the size of oil droplets, pH, oxygen concentration, and chemical structures of lipids can all contribute to the impact on the oxidation of O/W emulsions (Figure 1) [25,26]. 

#### 2.2.1. Physicochemical Properties of the Interfacial Region

Physicochemical properties of the interfacial region can impact emulsion oxidation because they affect lipid hydroperoxides and their capability to interact with metal ions [28]. 

The thickness of the interfacial region can strongly affect the oxidation rate of emulsions because the lipid oxidation rate is influenced by the interactions of unsaturated lipids in the oil droplets with pro-oxidants present in the water phase. Therefore, inhibiting the transfer of pro-oxidants in the water phase toward the oil droplets can reduce lipid oxidation. This can be achieved by forming thick coatings around the oil droplets [22,29,30]. 

The charge of the interfacial region is another factor that can affect the oxidation rate of O/W emulsions. The charge of the interfacial region depends on surfactant type, other oppositely charged components in the emulsion that can attach to the emulsion droplets surface, ionic strength, and pH [31,32,33]. The negative charge of the interfacial region can enhance the oxidation rate by attracting metal ions towards the oil phase [34]. The pH of the emulsion can alter the charge of the proteins. The charge of the interfacial region stabilized by proteins is negative at pH values higher than the isoelectric point of proteins. Therefore, proteins can enhance the oxidation rate of emulsions at high pH values [35].

#### 2.2.2. Oil and Aqueous Phase Components

Free fatty acids that remain in the oil phase after being refined can affect the lipid oxidation rate. In this regard, Waraho et al. [16] stated that oleic acid reduced oxidative stability via enhancing the negative charge of emulsion droplets. The pro-oxidant effects of unsaturated fatty acids were in the order of linolenic < linoleic < oleic. This pro-oxidant effect is related to the ability of free fatty acids to attract pro-oxidant metal ions and to co-oxidize triacylglycerols in bulk oil [36]. 

Phospholipids can both inhibit and promote lipid oxidation in the oil phase. The pro-oxidant effects of phospholipids are due to their anionic physical structures that can attract pro-oxidant metal ions. Meanwhile, their inhibitory effect against lipid oxidation is due to their ability to increase the affinity of antioxidants to locate at the interfacial region, decompose hydroperoxides, and chelate metal ions [17]. 

Proteins in the water phase can decrease the oxidation rate in O/W emulsions using several different mechanisms. For example, cysteine, tryptophan, and tyrosine amino acids can exhibit radical scavenging and metal-chelating activities at pH values higher than the isoelectric point of the protein [28]. 

Some polysaccharides, such as glycoproteins, gum Arabic, and soluble soybean polysaccharides, can enhance O/W emulsion oxidative stability through interacting with metal ions and free radicals. In addition, polysaccharides can lower the diffusion rate of pro-oxidant metal ions and oxygen toward the interfacial region by enhancing the viscosity of the water phase [37]. 

#### 2.2.3. Size of Oil Droplets

The size of oil droplets can alter lipid oxidation rate by affecting the contact area between unsaturated fatty acids and pro-oxidants in the water phase. It is expected that small droplets can oxidize faster because they can create a larger contact area between unsaturated fatty acids and pro-oxidants in the water phase [38]. However, some researchers have indicated that O/W emulsions containing small droplets have higher oxidative stability than those containing larger droplets [39]. These contradictory results show that adjusting the size of oil droplets can alter other parameters affecting the rate of lipid oxidation. For example, droplets of different sizes have different curvatures in the droplet surface, which can change the packing properties of the emulsifiers. In addition, when lipid concentration is constant, reducing the size of droplets can enhance the concentration of the interfacial emulsifier and decrease the distance between oil droplets. This reduction in the distance between oil droplets is expected to enhance the exchange of lipid oxidation products, transition metal ions, and antioxidants between oil droplets. [27]. Increasing the transfer rate of antioxidants between oil droplets can enhance their efficiency in O/W emulsion and reduce oxidation rate, while increasing the transfer rate of lipid oxidation products and transition metal ions can enhance the oxidation rate [40]. Taken together, reducing oil droplet size in different lipid systems does not always have the same effect on lipid oxidation. Depending on the type of lipid system and the molecular species present in the system, the reduction in oil droplets may decrease or increase the oxidation rate.

#### 2.2.4. Transfer of Oxidants and Antioxidants between Oil Droplets

Besides the importance of the physicochemical properties of the interfacial region, the transport of lipid oxidation products, transition metal ions, and antioxidants is a crucial event that is often ignored in the determination of the oxidation rate in emulsions. Three mechanisms of mass transfer in O/W emulsions include (I) diffusion, (II) collision–exchange–separation, and (III) micelle-assisted transfer. According to the diffusion mechanism, molecules diffuse from one oil droplet to another through the water phase. According to the collision–exchange–separation mechanism, when oil droplets collide with each other, they transfer from one particle to another. According to the micelle-assisted transfer mechanism, molecules solubilize in micelles within the water phase and then transfer between lipid droplets [25]. Three types of compounds can be produced during lipid oxidation based on their diffusion properties as follows: (I) water-soluble compounds, such as carbonyl compounds; (II) surface-active compounds (e.g., LOOHs); and (III) hydrophobic compounds (e.g., lipoperoxy radicals) [41]. Water-soluble compounds can transfer between oil droplets through the diffusion mechanism, while hydrophobic compounds can be transferred only through the collision–exchange–separation mechanism. Transfer of hydrophobic compounds through the collision–exchange–separation mechanism is slower than that of water-soluble compounds through the diffusion mechanism. When micelles are present in the water phase, the micelle-assisted transfer mechanism occurs that can result in a drastic increase in the transfer rate of hydrophobic compounds between oil droplets. The rate of collision–exchange–separation and micelle-assisted transfer mechanisms depends on the size and concentration of lipid droplets and micelles. In O/W emulsions that contain a high concentration of micelles and large droplets, the micelle-assisted transfer mechanism would be faster than the collision–exchange–separation mechanism [25]. The ability of molecules produced during lipid oxidation to transfer from one oil droplet to the other one depends on their stability in the system. For instance, peroxyl radicals induced by AMVN (2,2′-azobis (2,4-dimethylvaleronitrile) could transfer between oil droplets [42], while no transfer was observed for alkoxyl radicals produced by di-tertbutyl peroxide [43]. This could be related to the shorter life span of alkoxy radicals (10^−6^ s) than peroxyl radicals (0.5–7 s), which limits their transfer from one oil droplet to another one [42,43]. A peroxyl radical can cross much longer distances (0.14 mm in a non-viscous medium and 0.2 × 10^−3^ mm in a viscous one) than alkoxyl radicals (10^−4^ mm in a non-viscous medium and 10^−7^ mm in a viscous one) [25]. LOOH molecules have a higher half-life than their radical homologs (LOO•) and are able to transfer between oil droplets. During lipid oxidation, the formation of lipid hydroperoxides micelles in the water phase of O/W emulsions can shift the transfer mechanism of lipid oxidation products from collision–exchange–separation to micelle-assisted transfer, which results in a sudden transition from the initiation stage to the propagation stage. The initiation stage is a transfer-controlled rate, meaning that that the rate of lipid oxidation is limited only by how fast oxidants can transfer from the oxidized oil droplet to the other one. Meanwhile, the propagation stage is a reaction-limited rate, meaning that the time required for oxidants to encounter an oxidizable substrate suddenly becomes negligible compared to the time required for the reaction to occur. In the presence of excess amounts of surfactants in the system, competitive adsorption processes in mixed micelles between surfactants and LOOH molecules may occur. 

#### 2.2.5. Surfactant Micelles

When a surfactant is used at concentrations higher than its CMC value, surfactant micelles are formed in the water phase of emulsions. These micelles can rapidly exchange lipid hydroperoxides, antioxidants, and metal ions by interacting with other micelles, with the water phase, or with oil droplets. In addition, surfactant micelles can form co-micelles with other surface-active agents and change the partitioning of emulsion components among the interfacial region, oil droplets, and aqueous phase [44]. Surfactant micelles can contribute to the mass transport of oxidation products among the oil droplets; therefore, they can cause an increase or decrease in oxidation rate. In this regard, Raudsepp et al. [42] indicated that radicals that are produced inside oil droplets could contribute to the initiation of lipid oxidation in neighboring oil droplets when surfactant micelles exist in the solution. In some cases, surfactant micelles can reduce O/W emulsion oxidation [45]. It has been reported that surfactant micelles can partition iron out of the oil droplets, thereby reducing the oxidation rate [46]. Additionally, when surfactant micelles are present, lipid hydroperoxides can partition out of the oil droplets, thereby reducing the oxidation rate [44]. Furthermore, surfactant micelles can increase the efficiency of highly hydrophobic antioxidants by favoring their partitioning at the interfacial region [47].

#### 2.2.6. pH

The pH of the water phase can affect the charge of the oil droplets, the solubility of metal ions, and the chemical reactivity of antioxidants, metal ions, and chelators [14,48,49]. The lipid oxidation rate in emulsions containing tannic acid increased in response to a decrease in pH from 3 to 7, which can be attributed to the decrease in the antioxidant capacity of tannic acid where acidic pH exists. In addition, acidic pH can increase the solubility and activity of the metal ions in the water phase [50]. Furthermore, acidic pH can reduce the metal-chelating capacity of the tannic acid [48]. It has been suggested that anionic polysaccharides, pectin, and xanthan gum have higher antioxidant activity at pH 3.5 than at pH 7. Xanthan gum was a better antioxidant than pectin at pH 3.5, while it showed a pro-oxidant effect at pH 7. When pH is acidic, anionic polysaccharides can bind to cationic metal ions, thereby reducing the pro-oxidant effect of metal ions [51].

## 3. Recent Advances in Reducing the Oxidation Rate of Bulk Oil

### 3.1. Esterifying Antioxidants

Esterifying antioxidants can alter the hydrophilic–lipophilic balance and improve the efficiency of antioxidants in bulk oil. For instance, polydatin esters proved to be more effective than α-tocopherol in reducing the oxidation rate in fish oil [52]. In addition, glyceryl caffeate esters exhibited better efficiency than α-tocopherol in reducing the oxidation of bulk tuna oil [53]. The unsaturation degree of the alkyl chain in antioxidant esters can affect their antioxidant activity in bulk oil. For instance, hexanoate, caprylate, decanoate, dodecanoate, and palmitate esters of resveratrol were reportedly more effective than resveratrol in reducing conjugated dienes in bulk oil. However, eicosapentaenoate and docosahexaenoate esters of resveratrol produced the same or even higher amounts of conjugated dienes than the resveratrol itself because PUFAs that are connected to resveratrol can also be oxidized and form conjugated dienes [54]. The concentration of esterified antioxidants can also affect their efficiency in bulk oil. Zhong and Shahidi [55] reported that at lower concentrations, tetrastearate-epigallocatechin gallate showed higher antioxidant activity than hydrophilic analogue epigallocatechin gallate, although epigallocatechin gallate was more active at higher concentrations. The presence of association colloids can also affect the efficiency of esterified antioxidants in bulk oil. The water–oil interface of association colloids can affect the partitioning and activity of pro-oxidants and antioxidants in bulk oils. According to research, in the absence of DOPC association colloids, few differences were observed between the lag phase of conjugated dienes formation in bulk oil incorporated with chlorogenic acid and those of bulk oil incorporated with few butyl, hexadecyl, and dodecyl esters of chlorogenic acid. When DOPC association colloids were present, the lag phase of conjugated dienes production was in the order of free chlorogenic acid < butyl ester < dodecyl ester ∼ hexadecyl ester [20]. Some phenolic compounds can exert synergistic effects with their esterified derivatives. For instance, combinations of methyl gallate and gallic acid caused a longer induction period compared to each antioxidant alone. This indicates and describes the synergy contribution to the inhibition of chain initiation and/or propagation reactions and the likelihood of their promoted incorporations into the interfacial area of association colloids [56]. Additionally, sunflower oil samples containing eugenol + eugenyl acetate and eugenol and eugenyl butyrate exhibited better efficiency than eugenol alone, which could be related to the better accessibility of eugenol to the interfacial region of the reverse micelles when eugenyl acetate and eugenyl butyrate are present [57].

### 3.2. Combining Antioxidants with Surface-Active Compounds

Incorporating surface-active agents, such as phospholipids, into bulk oils can enhance the efficiency of antioxidants. Mansouri et al. [3] reported that lecithin could promote the efficiency of methyl gallate and gallic acid by favoring their incorporation into the interfacial region of reverse micelles. In addition, the efficiency of ferulic acid, ethyl ferulate, and γ-oryzanol increased in the presence of lecithin. The number and size of reverse micelles are likely to increase considerably when surface-active compounds are present, which can enhance the acceptance capacity of hydroperoxides in the structure of reverse micelles. Polar antioxidants tend to concentrate at the interfacial region of reverse micelles. Therefore, more interactions can occur between hydroperoxides and antioxidants. In addition, increasing the number of reverse micelles is expected to excite the movement of the non-polar parts of antioxidants into the interfacial region of these structures [58]. 

## 4. Recent Advances in Reducing the Oxidation Rate of O/W Emulsions

### 4.1. Formation of Thick Coatings around the Oil Droplets

Forming thick coatings around oil droplets can inhibit oxidation by inhibiting the movement of pro-oxidants present in the water phase toward the unsaturated lipids in the oil droplets [38]. The size of surfactants, the conformation of their head and tail groups, or conditions where biopolymers create layers around the oil droplets can affect the thickness of the interfacial region [33]. Some effective strategies for enhancing the thickness of the interfacial membrane include, but are not limited to, enhancing the layers of the interfacial membrane, applying Pickering particles to stabilize the emulsion, and applying complexes or conjugates of biopolymers at the interfacial layers. 

#### 4.1.1. Enhancing the Layers of the Interfacial Membrane 

Multilayer O/W emulsions are generated through the electrostatic deposition technique. Selected examples of multilayer emulsions that have been produced with the aim of inhibiting lipid oxidation at the interfacial region are presented in Table 2. Katsuda et al. [59] reported that emulsion containing citrus pectin and β-lactoglobulin exhibited higher oxidative stability than the emulsion that was stabilized merely with β-lactoglobulin. They suggested that the pectin layer limited interaction between unsaturated fatty acids and metal ions. In addition, anionic pectin can bind with metal ions. The branched structure of pectin can create a layer with higher thickness and cause a greater steric effect than other linear anionic polymers [60]. Additionally, when flaxseed O/W emulsion was produced by sodium caseinate and pectin, it had a lower peroxide value (PV) and TBARS value than emulsions produced by sodium caseinate alone during 22 days of storage at 55 °C [61]. 

Emulsions containing three layers of surfactants are commonly produced to reduce the oxidation rate of emulsions. Xu et al. [67] prepared O/W emulsions produced by three layers of chitosan, flaxseed gum, and whey protein isolate. They observed that the presence of chitosan in the third layer enhanced the thickness of the interfacial membrane, scavenged free radicals, and repelled metal ions. The oxidative stability of emulsions with three layers of emulsifiers is not always higher than those emulsions with two layers of emulsifiers. The emulsion stabilized with three layers of Citrem/chitosan/alginate showed a higher oxidation rate than the emulsion stabilized with two layers of Citrem/chitosan, although the former exhibited a lower oxidation rate than the emulsion stabilized with one layer of Citrem. This could be due to the positive charge of oil droplets coated by Citrem/chitosan, which can repel metal ions [60]. 

In addition, the thickness and charge of the interfacial region should also be considered. Research has suggested that emulsions with inner whey protein isolate and outer fish gelatin layers show higher oxidative stability than those with an inner fish gelatin layer and an outer protein isolate layer [68]. This can be attributed to better interfacial activity of the whey protein isolate that leads to higher interface adsorption than fish gelatin, even as positive charges of the outer fish gelatin layer electrostatically repelled the metal ions. 

#### 4.1.2. Applying Pickering Particles to Stabilize Emulsions

Emulsions produced by solid particles (Pickering emulsions) have shown high physical and oxidative stability during the storage period [69]. Some Pickering emulsions are produced to inhibit lipid oxidation at the interfacial region (Table 3). The interfacial layer thickness of Pickering emulsions (ranging from 10 nm to 100 μm) is higher than surfactant-based emulsions (1~50 nm). Kargar et al. [70] reported higher oxidative stability of a Pickering emulsion produced by silica particles, compared to an emulsion produced by Tween 20, which may be related to the higher thickness of the interfacial layer of emulsion produced by silica particles. Furthermore, microcrystalline cellulose Pickering particles provided higher stability against oxidation, compared to modified starch. The antioxidant activity of microcrystalline cellulose was related to a combination of free radical scavenging and the generation of a thick coating around the oil droplets [69]. Furthermore, using fish oil in the form of Pickering emulsion produced by chitosan-stearic acid nanogels inhibited mayonnaise oxidation [71]. 

**Figure 2:**
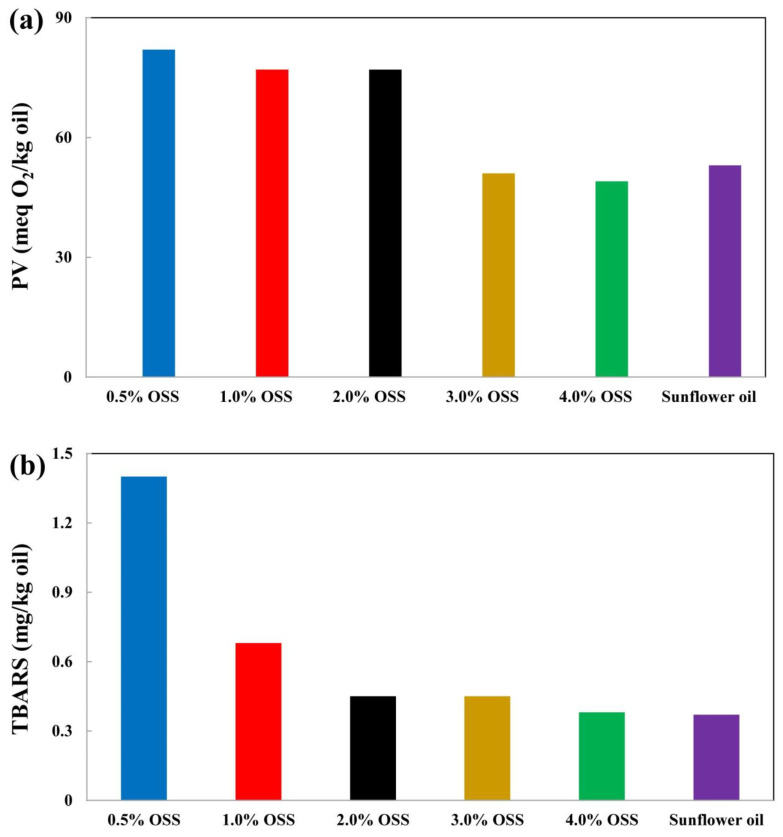
(**a**) Peroxide value (PV); (**b**) thiobarbituric acid reactive substances value (TBARS) of Pickering emulsions with different octenyl succinic anhydride modified starch particle (OSS) concentration after 14 days of storage at 50 °C (adopted from Ref. [5]).

The oxidation rate of the Pickering emulsion reduces when Pickering particle concentration is enhanced. For instance, the oxidation rate of emulsions produced by hydrophobically modified starch particles was effectively enhanced in response to higher concentrations of starch particles that increased from 0.5% to 4.0% through an entire incubation period of 14 days storage at 50 °C (Figure 2) [5]. In addition, when the microcrystalline cellulose concentration increased in the system, the thickness of the surface layer around the oil droplets increased but so did the concentration of non-absorbed cellulose microcrystalline particles enhanced in the water phase, thereby leading to the formation of a continuous cellulose microcrystalline network. This network in the water phase can reduce oxidation by slowing down the mobility of pro-oxidants in the water phase. This possibly occurs due to an increase in the viscosity of the system [69]. Complex particles have reportedly shown an enhanced capability to stabilize Pickering emulsions compared to particles with a single ingredient. For example, Zhang et al. [83] reported that emulsions produced by pure soy protein isolates exhibited remarkably higher PV than emulsions produced by soy protein isolate-cellulose nanofibril complexes. Additionally, emulsions produced by adding a secondary layer of chitosan to an existing O/W emulsion of faba bean protein isolates showed a thicker interfacial layer than the O/W emulsion produced by faba bean protein isolates alone [84].

### 4.2. Entrapping Emulsified Oil Droplets in a Gel Matrix

**Figure 3:**
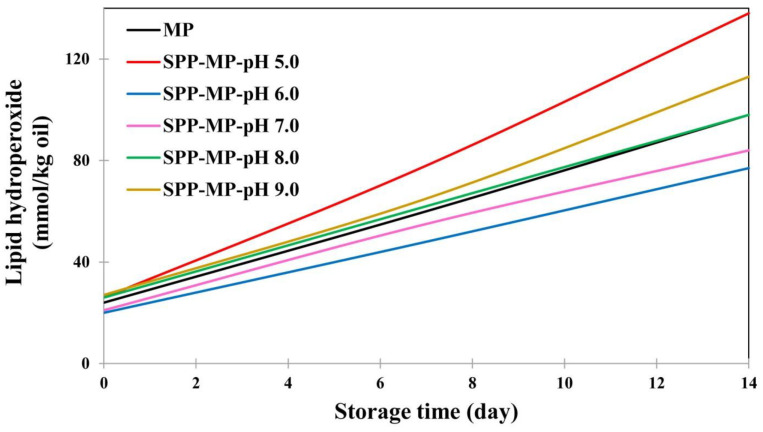
Changes in lipid hydroperoxides of sodium pyrophosphate (SPP)—modified myofibrillar protein (MP) emulsion gels under different pH conditions during 14 days of storage at 25 °C (adopted from Ref. [78]).

Emulsion gels consist of emulsified oil droplets that are entrapped in a gel network. Emulsion gels are produced via the addition of thickeners into the emulsions. Furthermore, emulsion gels can be produced by adding cross-linking biopolymers [85,86]. Some emulsion gels are reportedly produced to inhibit lipid oxidation at the interfacial region (Table 3). Emulsion gels exhibit higher oxidative stability than conventional emulsions. In a relevant study, the PV of nanoemulsion gels that were based on a carbomer polymer decreased significantly compared to non-gelled linseed oil nanoemulsions. The high oxidative stability of emulsion gels can be explained by the fact that the enhanced viscosity of the water phase reduces the mobility of pro-oxidants during oxidation. In addition, emulsion gels that are prepared by cross-linking biopolymers can encapsulate PUFAs, thereby protecting them against oxidation [87]. The hydrocolloid type and its ways of structuring emulsion gels can affect the oxidative stability in hydrocolloids. When an emulsion gel is produced by a mixture of alginate and gelatin, it tends to exhibit higher oxidative stability than emulsion gels that result from only one biopolymer [88]. This increase in oxidative stability in the presence of an alginate and gelatin mixture can be explained by distinct mechanisms. Alginate can enhance the continuous phase viscosity that results in a delay in the diffusion of oxygen within the emulsion gel. On the other hand, gelatin, which is adsorbed onto the interfacial membrane, can act as a physical barrier and separate pro-oxidant compounds (in the water phase) from PUFAs in the oil phase. A mixture of gelatin and alginate imparted both of the said antioxidative mechanisms to the emulsion, which resulted in enhanced oxidative stability of the emulsion gel [88]. In addition, modifications in the phosphorylation of myofibrillar protein by sodium pyrophosphate reduced the oxidation rate of the emulsion gel at pH 6 and 7, compared to the unmodified emulsion gel (Figure 3). This improvement could be related to increased protein-protein cross-links through ionic interactions between phosphate groups and –NH^3+^ of amino acids, which formed homogeneously among absorbed and/or unabsorbed proteins, entrapping fractions of myofibrillar protein within the network. Thus, the oil droplets were better adhered to the gel network [89]. In fact, the gel matrix between the absorbed and unabsorbed protein at pH 6 and 7 can act as a physical barrier that can separate the pro-oxidant compounds present in the water phase from lipid substrates or by retarding the diffusion of oxygen within the emulsion gel [88,90]. In addition to the biopolymer type, different heating treatment methods can impact emulsion gel oxidation. For instance, microwave pretreatment reduced the oxidation rate of the emulsion gel produced by ovalbumin, inulin, and carrageenan, compared to emulsion gels produced without microwave pretreatment. This could have resulted from the fact that microwave pretreatment can increase interactions among ovalbumin, inulin, and carrageenan. In effect, it caused improvements in the encapsulation efficiency of the emulsion gel during emulsification and the gel formation processes, thereby enhancing the oxidative stability of the emulsion gel [91]. In contrast, ultrasonic pretreatment increased the PV and TBARS value of emulsion gels produced by rice bran oil and inulin, compared to rice bran oil itself. This increase in oxidation rate had roots in extreme physical forces that occurred during ultrasonic treatment. In fact, the said forces can decompose molecules and generate several highly reactive radicals during ultrasonic treatment [92].

### 4.3. Applying Surface-Active Biopolymers with Antioxidant Property

Surface-active biopolymers that are adsorbed at the interfacial layer can influence the rate of oxidation. The chemical composition of the interfacial region is an important parameter that can affect the rate of oxidation in O/W emulsion. Therefore, altering the chemical composition of the interfacial region can improve the oxidative stability of emulsions. Utilizing surface-active biopolymers with antioxidant activity can be used as an effective approach to inhibit oxidation at the interfacial region [31]. Several surface-active compounds have had frequent roles in inhibiting oxidation at the interfacial region of O/W emulsions (Table 4).

#### 4.3.1. Proteins and Peptides

Proteins are surface-active biopolymers, which can be adsorbed on oil droplet surfaces. Apart from physically stabilizing emulsions, some amino acids in food proteins exhibit metal-chelating and free radical scavenging activities. Proteins with methionine, cysteine, and tryptophan groups showed significant radical scavenging activity [28]. Numerous proteins have been proven to exhibit antioxidant activity in emulsions, including casein [90], whey proteins [92], and soy proteins [94].

Recently, the interfacial activity and antioxidant activity of amphiphilic peptides have gained substantial attention. Hydrolyzed proteins or peptides can inhibit oxidation scavenging free radicals and chelating metal ions [94]. Amphiphilic peptides can concentrate at the interfacial region and form a coating with a specific thickness to inhibit unsaturated lipids and pro-oxidant compounds from coming into direct contact with each other. These peptides must have a certain molecular size to exhibit amphiphilic properties. This is achievable via controlled hydrolysis of proteins. As such, the hydrolysis degree can range from 1 to 4. Forming an integral and rigid membrane from small peptides is difficult. To solve this issue, surfactants such as Tween 20 can be applied as co-emulsifiers with peptides. Adding proteins and peptides as co-emulsifiers with Tween 20 significantly reduced the rate of oxidation in O/W emulsion (*p* < 0.05) [108]. For instance, soy proteins and their hydrolysates exhibited significant emulsifying power and chemically stabilized the emulsions for two weeks of storage. The oxidation of proteins and peptides affected neither the physical stability of emulsions significantly, nor the peptide adsorption at the interfacial region. In addition, Zhang et al. [83] reported that using ultrasonic treatment can enhance the antioxidant capacity of rice peptide nanoparticles by enhancing the free radical scavenging by 3.5 fold and by boosting the metal-ion-chelating activity by 3.8 fold. Rice peptide nanoparticles can produce Pickering emulsions with high oxidative stability during the storage period. This could be related to antioxidant properties and the physical barrier effects of rice peptide nanoparticles.

#### 4.3.2. Polysaccharides

Polysaccharides can generate relatively thick hydrophilic coatings around the oil droplets. Thus, polysaccharides can generate strong long-range steric repulsions [28]. Polysaccharides can reduce the oxidation rate of emulsions where they are present at droplet surfaces. For instance, when arabinoxylan is sourced from corn bran, wheat bran, rye bran, or rice bran, it can be adsorbed on the surface of the oil droplets and thus reduce PV and TBARS concentration in the emulsions. However, arabinoxylan from rice bran was slightly more effective [109]. Some polysaccharides, such as beet pectin, contain antioxidant groups, such as ferulic acid and protein groups. Nevertheless, emulsions stabilized by beet pectin are highly sensitive to oxidation, probably due to residual metal ions associated with beet pectin [28].

#### 4.3.3. Protein-Polysaccharide

Emulsions that are produced from proteins alone are susceptible to creaming, phase separation, and coalescence when encountering adverse environmental conditions, such as changes in pH value, high ionic strength, and heat treatment. The role of proteins-polysaccharides as surfactants can help reduce the effects of environmental stress [110]. There are three categories of proteins-polysaccharides complexes, namely (a) Maillard conjugates, which are produced by the interaction of the amine group on a protein that has a reducing end of a polysaccharide, thereby generating a covalent bond; (b) electrostatic complexes that form between a protein and a polysaccharide with opposite net charges; and (c) naturally occurring complexes in which protein residues are covalently bound to polysaccharide chains [111]. When protein-polysaccharide conjugates are produced through a controlled Maillard reaction, they acquire a high level of antioxidant activity against oxidation. The improved ability of conjugates helps prevent oxidation, which could be related to the fact that some compounds produced during the Maillard reaction have strong antioxidant activity [112]. Pea protein isolate-gum Arabic conjugates showed a slight inhibitory effect against lipid hydroperoxide formation [83]. The greatest inhibitory effect occurred against hexanal production. The relevant research showed that conjugates formed a barrier that prevented hydroperoxides from interacting with transition metal ions, rather than serving as a scavenger to quench free radicals during the storage period. In addition, the conjugated mixture of whey protein–pectin was more effective than their unconjugated mixture in enhancing the oxidative stability of β-carotene at pH 7. The generation of a thicker interfacial layer via conjugates could significantly affect metal ions and free radicals in the water phase, thereby reducing their ability to degrade β-carotene [113]. Relevant to this context, the antioxidant activity of ovalbumin was significantly improved when it was covalently attached to dextran or galactomannan via the Maillard reaction [114,115].

#### 4.3.4. Protein-Polyphenol

Interactions of proteins and polyphenols primarily result from non-covalent (protein-polyphenol complex) or covalent bonds (protein-polyphenol conjugate) [116]. In the case of protein-polyphenol complexes, a protein molecule is physically attached to a polyphenol molecule. These complexes can form by electrostatic and hydrogen bonding, as well as hydrophobic interactions. Proteins can covalently link to polyphenols via alkaline, free radical, and enzymatic methods. Alkaline methods are reportedly capable of promoting extensive cross-linking between proteins. Enzymatic methods are the most specific methods for generating conjugates. This method is environmentally friendly, but the cost of enzymes is usually high. Free radical methods, such as ascorbic acid/H_2_O_2_ redox pair through the radical initiator reaction, can be applied to economically make protein-polyphenol conjugates In fact, the type of conjugates that arise from free radical methods have better antioxidant capacity than those produced by alkaline methods, probably due to the formation of more active sites on protein surfaces in free radical methods. In turn, they can interact with polyphenols and cause higher antioxidant activity [117].

Numerous studies have suggested that protein-polyphenol conjugates or complexes are able to show stronger antioxidant activity than original proteins. For instance, one study considered a whey protein isolates-lotus *proanthocyanidins* conjugate, which was sourced from seedpod plants. This conjugate exhibited higher antioxidant activity than a whey protein isolate when examined through DPPH and FRAP (ferric reducing antioxidant power) assays. In addition, it showed higher thermostability and better efficiency in producing flaxseed oil emulsions, compared to the efficiency of pure whey protein isolates. At the end of the storage period, low amounts of oxidation products are produced because of the use of the conjugate, compared to using protein alone in the O/W emulsion. Contrary to the uncombined polyphenol, conjugated protein-polyphenol molecules formed a dense layer at the interfacial region, which could increase the physical barrier effect [104]. According to Sun et al. [118], conjugating proteins with polyphenols enhanced the free radical scavenging activity, especially in the case of egg white protein-chlorogenic acid conjugates. The egg white protein-catechin and egg white protein-chlorogenic acid conjugates had FRAP values approximately 2.4- and 4.9-folds higher than that of the unmodified egg white protein [118]. In addition, polyphenols exhibited synergistic effects with protein hydrolysate when conjugates were formed [119]. Furthermore, polyphenols can increase surface activity when emulsified with protein hydrolysates, significantly enhancing their concentration at the interfacial area of the emulsions [120]. The emulsifying stability and oxidative stability of myofibrillar-protein-prepared O/W emulsions were improved by zein hydrolysates when associated with sage extract at certain concentrations. This improvement can be attributed to the increase in the adsorption of protein onto the oil–water interface in the presence of sage extract. In addition, the interfacial region was more compact in the emulsion with sage extract [121].

The interaction between proteins and polyphenols can reduce the pro-oxidant effect of polyphenols. In this regard, Dai et al. [122] reported that the antioxidant capacity of B-type procyanidin dimer was increased when rice protein was present. They stated that when the B-type procyanidin dimer interacts with rice glutelin via hydrophobic forces, there is less chance for pro-oxidant compounds to interact with polyphenols.

#### 4.3.5. Protein-Saponin

Saponins are surface-active compounds because they contain hydrophilic regions, such as xylose, rhamnose, galactose, arabinose, glucuronic acid, and fucose, as well as hydrophobic regions, such as gypsogenic acid and quillaic acid, on the same molecule. Saponins can generate a self-assembling 3D network of nanofibers that can stabilize Pickering emulsions [123]. Protein-saponin coatings inhibited protein and lipid oxidation more efficiently compared to protein coatings alone. Producing emulsions with a mixture of almond protein isolate-camellia saponin showed higher resistance to droplet flocculation compared to those produced by almond protein isolate alone after incubation at 45 °C for 0, 3, and 6 days. In addition, almond protein isolate-camellia saponin complexes were adsorbed onto the interfacial region and acted more effectively as interfacial antioxidants compared to the almond protein isolate. This stronger antioxidant activity of the almond protein isolate-camellia saponin complex could be related to the fact that the adsorbed camellia saponin molecules pose free radical scavenging and metal-ion-chelating effects. In addition, the almond protein isolate-camellia saponin possibly has the ability to create a physical barrier that sterically prevents metal ions from reaching the emulsified lipids [124].

#### 4.3.6. Protein-Polysaccharide-Polyphenol

Polysaccharides, polyphenols, and proteins can be assembled into ternary complexes with functional properties that combine the favorable effects of each compound. Covalent complexes of proteins-polysaccharides-polyphenols need heating or the addition of enzymes or chemicals. Non-covalent protein-polyphenol-polysaccharide complexes can be made available through mild processes [125]. It has been reported that the lactoferrin-hyaluronic acid-epigallocatechin gallate ternary complex could successfully produce soluble complexes at pH > 4, microgel dispersions at pH 3, and macroscopic hydrogels at pH 2. The antioxidant activity of the ternary complexes at pH 5 was much higher than at pH 3. This was related to the ability of the hydrogels to prevent free radicals in the water phase from accessing epigallocatechin gallate trapped inside [125]. It has been suggested that ternary complexes of zein-carboxymethyl chitosan-tea polyphenols could be applied as a useful material to stabilize emulsion to enhance β-carotene stability in functional foods because of its higher stability against acidity, heat, UV light, and ionic strength, compared to single protein and polysaccharide-protein systems, according to Ba et al. [106]. More research on the ternary complex of protein-polysaccharide-polyphenol revealed that the ternary complex showed an increased capacity to stabilize β-carotene emulsions compared to the capacity of binary conjugates to do the same. It has been reported that food processing treatments (e.g., microwave treatment and high-pressure homogenization) can increase the antioxidant capacity of β-glucan-soy protein hydrolysates-ferulic acid complexes. It is supposed that these treatments could be conducive to generating intermediate reductone compounds. These compounds can terminate free radical chain reactions [126].

### 4.4. Increasing the Fraction of Antioxidants at the Interfacial Region

Antioxidants exhibit higher effectiveness when they are distributed evenly at the interfacial area [127] where lipid radical production mainly occurs. Accordingly, localizing antioxidants at the interfacial region can increase their efficiency in reducing the oxidation rate of O/W emulsions. Several treatments, such as lipophilizing hydrophilic antioxidants, conjugating antioxidants with surfactants, and incorporating antioxidants into Pickering particles, can be proposed as effective methods for increasing the portion of antioxidants at the interfacial region.

#### 4.4.1. Lipophilization of Hydrophilic Antioxidants

According to the “polar paradox” theory, non-polar antioxidants tend to accumulate at the interfacial region, while polar antioxidants tend to locate themselves in the water phase. Accordingly, covalent modification of hydrophilic antioxidants through lipophilization can improve their efficiency in O/W emulsions. The lipophilization of antioxidants can be achieved through the esterification of their carboxyl groups with fatty alcohols or by esterifying their hydroxyl groups with fatty acids. Recent results in the available literature have considered a series of lipophilized antioxidants with different alkyl chain lengths that maximized the antioxidant efficiency, parallel to increasing the alkyl chain length, followed by a significant reduction in more hydrophobic lipophilized antioxidants. Known as the “cut-off” hypothesis, this parabolic dependence of antioxidant activity is on alkyl chain length for up to a critical chain length only, after which the antioxidant activity reduces [128]. The “cut-off” effect has been observed in the case of hydroxytyrosol esters [129,130], chlorogenic acid esters [131], and rosmarinic acid esters [132]. Three mechanisms of action for the “cut-off” effect are the “reduced mobility”, “internalization”, and “self-aggregation” hypotheses. According to the reduced mobility hypothesis, the diffusivity of the lipophilized antioxidants and their tendency to disperse at the interfacial region gradually slows down as the length of the hydrophobic chain decreases. The internalization hypothesis states that enhancing the alkyl chain length can drive the antioxidants away from the interfacial region and into the oil droplets core. The self-aggregation hypothesis states that antioxidants with longer alkyl chain lengths tend to form separate aggregates that reduce their availability to the interfacial region [2]. Alemán et al. [133] used “reduced mobility”, “internalization”, and “self-aggregation” hypotheses to explain the antioxidant activity of caffeic acid esters with different alkyl chain lengths in mayonnaise enriched with fish oil, as well as in milk enriched with fish oil emulsions. They observed that in the mayonnaise, medium alkyl chain length esters of caffeic acid (butyl, octyl, and dodecyl) showed higher oxidative stability than short (methyl) or long (octadecyl) alkyl chain length esters of caffeic acid. In contrast, in the milk, the most effective esters of caffeic acid were those with a short alkyl chain length (methyl and butyl). They stated that the high viscosity of mayonnaise could render diffusion harder among antioxidants in the medium. This decreases their capacity to move toward the interfacial layer. Therefore, according to the “reduced mobility” hypothesis, the medium alkyl chain length esters of caffeic acid, which can be positioned in the vicinity of the interfacial layer, would be more effective than short and long alkyl chain length esters of caffeic acid that need to move toward the interfacial layer. Conversely, because milk is much less viscous than mayonnaise, the mobility of caffeic acid esters with different alkyl chain lengths cannot be affected much. In addition, the higher polarity of milk emulsion than mayonnaise can allow caffeic acid esters with higher polarity to diffuse easier in the milk emulsion than in the mayonnaise emulsion, resulting in better efficiency of short chain esters of caffeic acid in milk emulsions. According to the “internalization” hypothesis, the mayonnaise emulsion, which has a higher oil content than the milk emulsion is a better “host” for long alkyl chain length esters of caffeic acid than the milk droplets. Long alkyl chain length esters of caffeic acid may self-aggregate (third hypothesis) in the aqueous phase of the milk emulsion due to its lower oil content.

The type and concentration of surfactants, pH, and O:W ratio can impact the efficiency of lipophilized antioxidants in an O/W emulsion. According to Sørensen et al. [134], caffeic acid and its esters (C1–C20) showed better efficiency in O/W emulsions that had been produced with Tween 20 than in those produced with Citrem. This difference was related to the interactions between lipophilized antioxidants and emulsifiers. For example, emulsifiers can change the partitioning of antioxidants in the emulsion. Furthermore, emulsifiers can form hydrogen bonds with antioxidants. It has been reported that the percentage of hydroxytyrosol and its lipophilic esters at the interfacial area can increase by first elevating the surfactant volume fraction. Furthermore, at a constant surfactant volume fraction, the interfacial molarity of hydrophilic antioxidants usually increases in response to an increase in the O:W ratio, whereas the opposite was observed in the case of most hydrophobic antioxidants (C6–C16). Regarding hydroxytyrosol esters that have medium hydrophobicity (C2–C4), changing the O:W ratio can lead to small changes in the interfacial concentration of these antioxidants [130]. Pertinently, da Silveira et al. [135] reported that when a negatively charged surfactant (sodium dodecyl sulfate) was used at pH 3, an emulsion containing butyl ester of sinapic acid showed higher malondialdehyde content and larger 2,4-decadienal values than those of the control emulsion. By increasing pH from 3 to 5, the pro-oxidant effect of sinapic acid butyl ester decreased. In addition, no pro-oxidant effect of sinapic acid butyl ester was observed when a surfactant with a positive charge (dodecyl trimethyl ammonium bromide) was applied, regardless of pH level. Accordingly, eliminating endogenous iron from the interfacial region through electrostatic repulsion or by reducing its solubility (pH 7) changed the intensity by which butyl ester in sinapic acid acted towards this ion [135]. Surfactant micelles can also affect the activity of lipophilized antioxidants in emulsions. In the absence of surfactant micelles, eicosyl rosmarinate was less effective in reducing emulsion oxidation than those of butyl, octyl, and dodecyl rosmarinate esters. When surfactant micelles were present, the efficiency of eicosyl rosmarinate increased significantly, while the efficiency of butyl and dodecyl rosmarinate esters decreased slightly. Previous studies on partitioning revealed that surfactant micelles enhanced eicosyl rosmarinate concentration at the interfacial area of the surfactant micelles and/or of the oil droplets [47]. Furthermore, da Silveira et al. [136] investigated the effects of surfactant micelles and the mode of their addition (e.g., pre-homogenization or post-homogenization) on the antioxidant activity of gallic acid esters in O/W nanoemulsions. When surfactant micelles were absent, propyl and palmitoyl gallate showed higher levels of efficiency than gallic acid, as well as in comparison to its octyl and dodecyl esters when added during pre-homogenization. This suggests that the mode of addition altered the partitioning of gallic acid esters. Contrariwise, when surfactant micelles were present, gallic esters could be added into any phase without loss of efficiency.

#### 4.4.2. Conjugating Antioxidants with Surfactants

Conjugating antioxidants with surfactants can increase their efficiency in O/W emulsions. For instance, when caffeic acid was conjugated to a surfactant or was covalently linked to a phosphatidylcholine, it reduced the oxidation rate of fish O/W emulsion more than what free caffeic acid did. This increase in oxidative stability was attributed to a closer vicinity between phenolic acid and the interfacial region [137]. Additionally, Yesiltas et al. [138] observed the production of emulsions with modified diacetyl tartaric acid esters of monoglycerides and diglycerides (DATEMs) with different alkyl chain lengths (C12 or C14) and with covalently linked caffeic acid. As such, these emulsions showed higher oxidative stability compared to those produced by commercial DATEM and free caffeic acid. This superiority confirmed the advantage of having antioxidants covalently linked to the surfactant. Similar results have been reported for mayonnaise enriched with fish O/W emulsion, which was also produced by modified DATEM with C14 and by covalently attached caffeic acid [139].

#### 4.4.3. Entrapping Antioxidants into the Pickering Particles

A new approach to locate antioxidants at the interfacial region is to entrap antioxidants in Pickering particles. In this method, antioxidants are forced to concentrate at the interfacial region, where their antioxidant activity can be maximized. In particular, Pickering emulsions that were produced by a modified β-cyclodextrin-β-carotene inclusion complex showed lower PV than those Pickering emulsions produced via a physical mixture of modified β-cyclodextrin, especially because the latter has succinic anhydride and β-carotene in its structure. This could be related to the accumulation of β-carotene at the oil–water interface through the inclusion complex [140]. In addition, Schröder et al. [141] reported that the production of both aldehydes and conjugated dienes, along with lipid hydroperoxides, was significantly smaller in the Pickering emulsions comprising carnosic acid and α-tocopherol in the colloidal lipid particles, compared to those Pickering emulsions comprising these antioxidants in oil droplets. The storage period in any case was 14 days at 25 °C.

## 5. Conclusions and Future Perspectives

Recent approaches to enhancing the oxidative stability of oil-based food products have occurred by reducing oxidation at the interfacial region. Esterifying natural antioxidants can be applied as an efficient approach to change their polarity and, as a consequence, their concentration at the interfacial region of association colloids in bulk oil. In addition, combining antioxidants with surfactants can prioritize the incorporation of antioxidants into the interfacial area of association colloids. In O/W emulsions, increasing the layers of the interfacial membrane and applying Pickering particles to stabilize an emulsion can inhibit peroxidation at the interfacial region by forming thick coatings around the oil droplets. In addition, entrapping emulsified oil droplets in a gel matrix can prevent the movement of metal ions into the interfacial region. This can be applied as a novel strategy for reducing the oxidation rate of emulsions. Furthermore, the oxidation rate of O/W emulsion can be reduced by modifying the composition of the interfacial region. This could be conducted by instilling antioxidant activity in covalent and/or non-covalent complexes of natural surface-active compounds, thereby making the complexes function as surfactants. In addition, localizing the antioxidants at the interfacial region of O/W emulsions can be performed by lipophilizing the hydrophilic antioxidants, conjugating antioxidants with surfactants, or entrapping antioxidants into Pickering particles. These can be proposed as effective strategies for reducing lipid oxidation at the interfacial region. More work is needed to determine the role of association colloids on the efficiency of antioxidants, with aims of designing more effective antioxidants in bulk oil. In addition, more research in this field could benefit future designing of O/W emulsion structures and the primary goal of obtaining emulsions with high levels of physical and oxidative stability that could assist in protecting PUFAs.

## Figures and Tables

**Table 1:** Pro-oxidant compounds in bulk oil and oil-in-water emulsion.

Pro-Oxidant Compound	Mechanism of Pro-Oxidant Effect	Reference
Transition metals (copper and iron)	Decomposing lipid hydroperoxides into free radicals	Mancuso et al. [14]
Singlet Oxygen	Directly attaching to the double bonds of unsaturated fatty acids to form lipid hydroperoxides	Min and Boff [15]
Free fatty acids	Attracting transition metals to the oil droplet surface when the pH of the emulsion is higher than the pK_a_ of free fatty acids	Waraho et al. [16]
Phospholipids	Attracting transition metals to the emulsion droplet surface	Chen et al. [17]
Ascorbic acid	Donating an electron to Fe^3+^ to form Fe^2+^ [18]	Jayasinghe et al. [18]
Carotenoids	Reaction of carotene peroxy radical with ^3^O_2_ and lipid molecules to produce lipid alkyl radicals	Iannone et al. [19]

**Table 2:** Multilayer emulsions inhibited lipid oxidation at the interfacial region.

Oil Type	First Layer	Second Layer	Third Layer	Oxidation Test	Result	Reference
Flaxseed Oil	Sodium caseinate	Pectin	-	PV and TBARS *	Positive effect of multilayer structure on improving physical and oxidative stabilities of conventional emulsions	Kartal et al. [61]
Chia oil	Modified sunflower lecithin	Chitosan	-	PV and TBARS	Higher oxidative stability of double-layer emulsions than monolayer ones	Julio et al. [62]
Fish oil	β-lactoglobulin or its hydrolysates	Pectin	-	PV	High oxidative stability of bilayer microcapsules	Tamm et al. [63]
Fish oil	Lecithin	Chitosan	-	TBARS	Great protective effect of multilayered microcapsules against lipid oxidation	Jiménez-Martín et al. [64]
Corn oil	Silk fibroin	Beet pectin	-	PV and hexanal formation	Better oxidative stability of double-layer emulsion than monolayer one	Chen et al. [65]
Fish oil	Citrem	Chitosan	Alginate	PV and TBARS	Better oxidative stability of Citrem/chitosan than Citrem alone or Citrem/chitosan/alginate	Gudipati et al. [60]
Linseed oil	Bovine serum albumin	Polyarginine	Dextran sulfate or tannic acid	TBARS	Efficient protection of oil droplets against oxidation by multilayer shell containing tannic acid	Lomova et al. [66]

* PV = peroxide value; TBARS = thiobarbituric acid reactive substances value; CD = conjugated dienes; AV = *p*-Anisidine value.

**Table 3:** Pickering emulsions and emulsion gels inhibited lipid oxidation at the interfacial region.

Biopolymer Type	Oil Type	Oxidation Test	Result	Reference
**Pickering emulsion**				
Chitosan-stearic acid nanogel	Sunflower oil	PV and TBARS *	Higher oxidative stability of emulsion stabilized by chitosan-stearic acid nanogel than emulsion stabilized by Tween 80	Atarian et al. [72]
Octenyl succinic anhydride modified rice starch	Sunflower oil	PV and TBARS	High oxidative stability of emulsion at pH 6–7	Zhu et al. [5]
Silica particles	Sunflower oil	PV and AV	Higher oxidative stability of emulsions stabilized with silica particles than emulsions stabilized with surfactants alone	Kargar et al. [70]
Cellulose nanocrystals	Rice bran oil	PV	Cellulose nanocrystals inhibited the formation of hydroperoxides in unsaturated fatty acids of rice bran oil phase	Angkuratipakorn, et al. [73]
Flaxseed protein and mucilage	Flaxseed oil	PV and TBARS	High oxidative stability of emulsions stabilized by flaxseed protein and mucilage complex particles	Nasrabadi et al. [74]
Gliadin/chitosan	Corn oil	PV and TBARS	Lower content of primary oxidation products in Pickering high internal phase emulsions (HIPEs) than in bulk oil	Zeng et al. [75]
Corn-peptide-functionalized calcium phosphate	Algal oil	PV and hexanal and volatile components formation	Water-in-oil-in-water Pickering emulsion stabilized by corn-peptide-functionalized calcium phosphate particles showed higher oxidative stability than emulsion prepared by bare calcium phosphate	Ruan et al. [76]
**Emulsion gel**				
Sodium caseinate	Sunflower oil	PV, hexanal, and 1-octen-3-ol formation	Higher oxidative stability of emulsion gel than conventional emulsion	Lim et al. [77]
Myofibrillar protein modified by sodium pyrophosphate	Soybean oil	PV	High oxidative stability of emulsion gels at pH 6 and 7	Chen et al. [78]
Carrageenan	Sunflower oil	TBARS and cholesterol oxidation products	Higher oxidative stability of burger patties containing emulsion gel as pork-back-fat replacer than control (burger patties containing pork back fat)	Poyato et al. [79]
Gelatin, alginate, or their mixture	Olive oil	PV and AV	Higher oxidative stability of alginate-gelatin mixed emulsions than systems produced with only one biopolymer	Sato et al. [80]
Ovalbumin, inulin, and carrageenan	Pomegranate seed oil	PV	High oxidative stability of emulsion gel	Li et al. [81]
locust bean, κ-carrageenan, xanthan, and maltodextrin	Flaxseed oil	PV, TBARS, and acid value	High oxidative stability of emulsion gels during storage period	Nasirpour-Tabrizi et al. [82]

* PV = peroxide value; TBARS = thiobarbituric acid reactive substances value; CD = conjugated dienes; AV = *p*-Anisidine value.

**Table 4:** Effects of surface-active compounds on inhibiting lipid oxidation at the interfacial region of the oil-in-water emulsions.

Surface-Active Compound	Oil Type	Results	Oxidation Test	Reference
**Proteins and peptides**				
β-Casein, β-lactoglobulin, and bovine serum albumin	Rapeseed oil	Lower efficiency of protein-stabilized interfaces than Tween 20 or Tween 80-stabilized interfaces at inhibiting lipid oxidation	Oxygen uptake, CD, and volatile compound formation *	Berton et al. [90]
Sodium caseinate	Walnut oil	Slow lipid oxidation but rapid protein oxidation of emulsions stabilized solely by sodium caseinate	PV and TBARS	Yi et al. [91]
Gliadin, sodium caseinate, and whey protein isolate	Fish oil	Higher efficiency of gliadin and whey protein isolate than sodium caseinate at inhibiting lipid oxidation	PV and TBARS	Qiu et al. [92]
Legume protein (lentil, pea, and faba bean)	Fish oil	Higher antioxidant activity of pea and faba bean proteins than whey proteins in washed emulsions	PV and TBARS	Gumus et al. [93]
Casein, whey protein isolate, and soy protein isolate	Corn oil	Higher antioxidant activity of casein than whey protein isolate and soy protein isolate	PV and headspace hexanal formation	Hu et al. [94]
Soy protein isolate	Soybean oil	Higher oxidative stability of emulsions treated with ultra-high-pressure homogenization than untreated emulsions	PV and TBARS	Fernandez-Avila and Trujillo [95]
Whey protein hydrolysate, soy protein hydrolysate, and blue whiting	Fish oil	Higher antioxidant activity of whey protein hydrolysate than soy protein hydrolysate and blue whiting	PV, AV, and volatile compounds	Padial-Domínguez et al. [96]
Cod bone peptides	Soybean oil	High oxidative stability of emulsions added with cod bone peptides	TBARS	Zhao et al. [97]
**Polysaccharides**				
Microcrystalline cellulose and modified starch	Sunflower oil	Higher oxidative stability of emulsions stabilized by microcrystalline cellulose than modified starch	PV and AV	Kargar et al. [69]
Modified starch and gum Arabic	Rice bran oil	Higher oxidative stability of emulsions containing modified starch than those emulsions containing gum Arabic	PV and hexanal formation	Charoen et al. [34]
Enzymatic degraded polysaccharides from *Enteromorpha prolifra*	Fish oil	High physical and oxidative stabilities of fish oil emulsion system (5% oil, 1% Enzymatic degraded polysaccharides, and 1% Tween 80)	PV and TBARS	Shi et al. [98]
**Proteins-polysaccharides**				
Whey protein isolate-gum Arabic	Conjugated linoleic acid	Nano-sized whey protein isolate/gum Arabic intramolecular soluble complexes significantly improved the oxidative stability of emulsions in comparison with individual protein or polysaccharide	Oxygen consumption measurement	Yao et al. [99]
Pea protein isolate-gum Arabic conjugate	Corn oil	Effective prevention of lipid oxidation by pea protein isolate-gum Arabic conjugates	PV and hexanal formation	Zha et al. [100]
Chitosan-peptide conjugate	Soybean oil	Higher antioxidant activity of chitosan-peptide conjugates than peptide, chitosan, and mixture of peptide-chitosan	TBARS	Meng et al. [101]
**Proteins-polyphenols**				
Duck egg albumen hydrolysate-epigallocatechin gallate conjugates	Fish oil	High efficiency of duck egg albumen hydrolysate in improving oxidative stability of emulsion	PV and TBARS	Quan and Benjakul [102]
Porcine bone protein hydrolysates-rutin conjugates	Soybean oil	Emulsions coated by Porcine bone protein hydrolysates and Porcine bone protein hydrolysates-rutin conjugates exhibited a high oxidative stability	PV and TBARS	Liu et al. [103]
Whey protein isolates-lotus seedpod proanthocyanin conjugate	Flaxseed oil	Higher antioxidant activity of conjugate than pure protein	CD and malondialdehyde	Chen et al. [104]
Pea protein-tannic acid complex	Flaxseed oil	High efficiency of pea protein-tannic acid complex in enhancing oxidative stability of emulsion	CD and TBARS	Li et al. [105]
**Proteins-saponins**				
Almond protein isolate-camellia saponin	Walnut oil	Higher resistant of almond protein isolate-camellia saponin-coated droplets to oxidation than almond protein isolate-coated droplets	PV and TBARS	Ba et al. [106]
Chickpea protein isolate-saponin isolated from ginseng	Antarctic krill oil	Ultrasound treatment improved antioxidant activity of chicken protein isolate-saponin isolated from ginseng complex	PV and TBARS	Xu et al. [107]

* PV = peroxide value; TBARS = thiobarbituric acid reactive substances value; CD = conjugated dienes; AV = *p*-Anisidine value.

## Data Availability

Data is contained within the article.
